# Coevolution Drives the Emergence of Complex Traits and Promotes Evolvability

**DOI:** 10.1371/journal.pbio.1002023

**Published:** 2014-12-16

**Authors:** Luis Zaman, Justin R. Meyer, Suhas Devangam, David M. Bryson, Richard E. Lenski, Charles Ofria

**Affiliations:** 1 BEACON Center for the Study of Evolution in Action, Michigan State University, East Lansing, Michigan, United States of America; 2 Department of Computer Science and Engineering, Michigan State University, East Lansing, Michigan, United States of America; 3 Department of Systems Biology, Harvard Medical School, Boston, Massachusetts, United States of America; 4 School of Medicine, Wayne State University, Detroit, Michigan, United States of America; 5 Microbiology and Molecular Genetics, Michigan State University, East Lansing, Michigan, United States of America; University of Lausanne, Switzerland

## Abstract

The evolution of complex organismal traits is obvious as a historical fact, but the underlying causes—including the role of natural selection—are contested. Gould argued that a random walk from a necessarily simple beginning would produce the appearance of increasing complexity over time. Others contend that selection, including coevolutionary arms races, can systematically push organisms toward more complex traits. Methodological challenges have largely precluded experimental tests of these hypotheses. Using the Avida platform for digital evolution, we show that coevolution of hosts and parasites greatly increases organismal complexity relative to that otherwise achieved. As parasites evolve to counter the rise of resistant hosts, parasite populations retain a genetic record of past coevolutionary states. As a consequence, hosts differentially escape by performing progressively more complex functions. We show that coevolution's unique feedback between host and parasite frequencies is a key process in the evolution of complexity. Strikingly, the hosts evolve genomes that are also more phenotypically evolvable, similar to the phenomenon of contingency loci observed in bacterial pathogens. Because coevolution is ubiquitous in nature, our results support a general model whereby antagonistic interactions and natural selection together favor both increased complexity and evolvability.

## Abstract 2

Experiments using a digital host-parasite model system show that coevolution can drive the emergence of complex traits and more evolvable genomes. Homepage Title: Parasitism Drives the Evolution of Complexity

## Author Summary

Over billions of years, life has evolved into the extraordinarily diverse and complex organisms that populate the Earth today. Although evolution often proceeds toward increasing complexity, more complex traits do not necessarily make organisms more fit. So when and why is greater complexity favored? One hypothesis is that antagonistic coevolution between hosts and parasites can drive the evolution of more complex traits by promoting arms races with increased defenses and counter-defenses. Here, by using populations of self-replicating host computer programs and parasitic programs, which steal processing power from their hosts, we demonstrated that coevolution promotes complexity and dissected how it does so. Instead of simple escalation, we found that a diversity of coevolving lineages must arise for coevolution to drive complex traits. Surprisingly, coevolution had a second effect; it promoted the evolution of more evolvable hosts. As a consequence, mutations in the evolved host genomes that confer resistance to parasites occur at high rates, which help the coevolved hosts outrun their parasites. Our experiments with an artificial system demonstrate how the naturally ubiquitous process of coevolution can promote complexity and favor evolvability.

## Introduction

Life emerged on Earth some 4 billion years ago and has evolved increasingly complex traits, including intricate biochemical pathways, elaborate developmental networks, and powerful neural architectures [1],[2]. However, the processes responsible for promoting this complexity remain poorly understood [1]–[9]. Is adaptation by natural selection largely responsible for this complexity and, if so, what is the nature of that selection? Or is this apparent trend an artifact that reflects the initial conditions and lower bounds to complexity? Given the limitations of historical data for answering these questions, experimental evolution offers an alternative approach to explore these issues and test specific hypotheses. However, the emergence of complexity in nature is a slow process, one not readily replicated in the laboratory [8],[10]; and without an objective way to measure the complexity of organismal traits [11],[12], rhetorical arguments may obscure and delay empirical research on this fundamental problem.

Fortunately, computational approaches have advanced beyond traditional numerical simulations, and it is now possible to test evolutionary hypotheses by running experiments with computer programs that self-replicate, mutate, compete, and evolve [13]. In one study, Lenski and colleagues [14] used the Avida [15] system to examine the role of selection for intermediate steps along many evolutionary paths to a particularly complex trait, the EQUALS (EQU) logic function. Because Avida is computational, the authors could readily observe changes over thousands of generations; moreover, the complexity of traits could be objectively quantified as the number of building blocks (in this case, NAND instructions) required for their execution. By allowing initially identical populations to evolve in different environments, Lenski and colleagues demonstrated that the most complex traits emerged only when simpler functions were also selectively favored, which promoted the accumulation of the necessary building blocks [14].

Here we use this system to ask whether coevolution—specifically, parasite-host interactions—can drive complexity to higher levels than would otherwise be achieved. Several authors, including Dawkins and Krebs [7] and Vermeij [16], have proposed that coevolutionary “arms races” lead to increased complexity as adaptations and counter-adaptations favor more and more extreme traits [6]. Indeed, we show that host-parasite coevolution produced substantially more complex host traits than did evolution in the absence of parasites. Moreover, we show that this complexity arose in the evolving computer programs, in part, by an unexpected process: selection for increased evolvability, which was achieved by genetic mechanisms reminiscent of so-called “contingency loci” that are found in many pathogenic bacteria [17].

In Avida, both host and parasite organisms are self-replicating programs that must expend CPU cycles to execute instructions in their genomes [18]. The genetic instruction set includes basic arithmetic and input/output operations as well as operations that allow storage and manipulation of binary numbers in temporary memory via a set of stacks. Coordinated execution of appropriate sets of instructions allows organisms to obtain resources (in the case of hosts) or infect hosts (in the case of parasites) and copy their genomes instruction-by-instruction to produce offspring. The copying process occasionally introduces mutations including point mutations, insertions, and deletions that may affect the progeny's phenotype. As in nature, most mutations are deleterious or neutral, but occasional beneficial mutations improve an organism's ability to acquire resources, infect hosts or resist parasites, or reproduce. These benefits may enable genotypes to increase in frequency as they displace less fit conspecifics because of their faster acquisition and more efficient use of CPU cycles. Thus, populations of digital organisms, like their counterparts in nature, typically evolve to better fit their environments [13].

**Figure 1:**
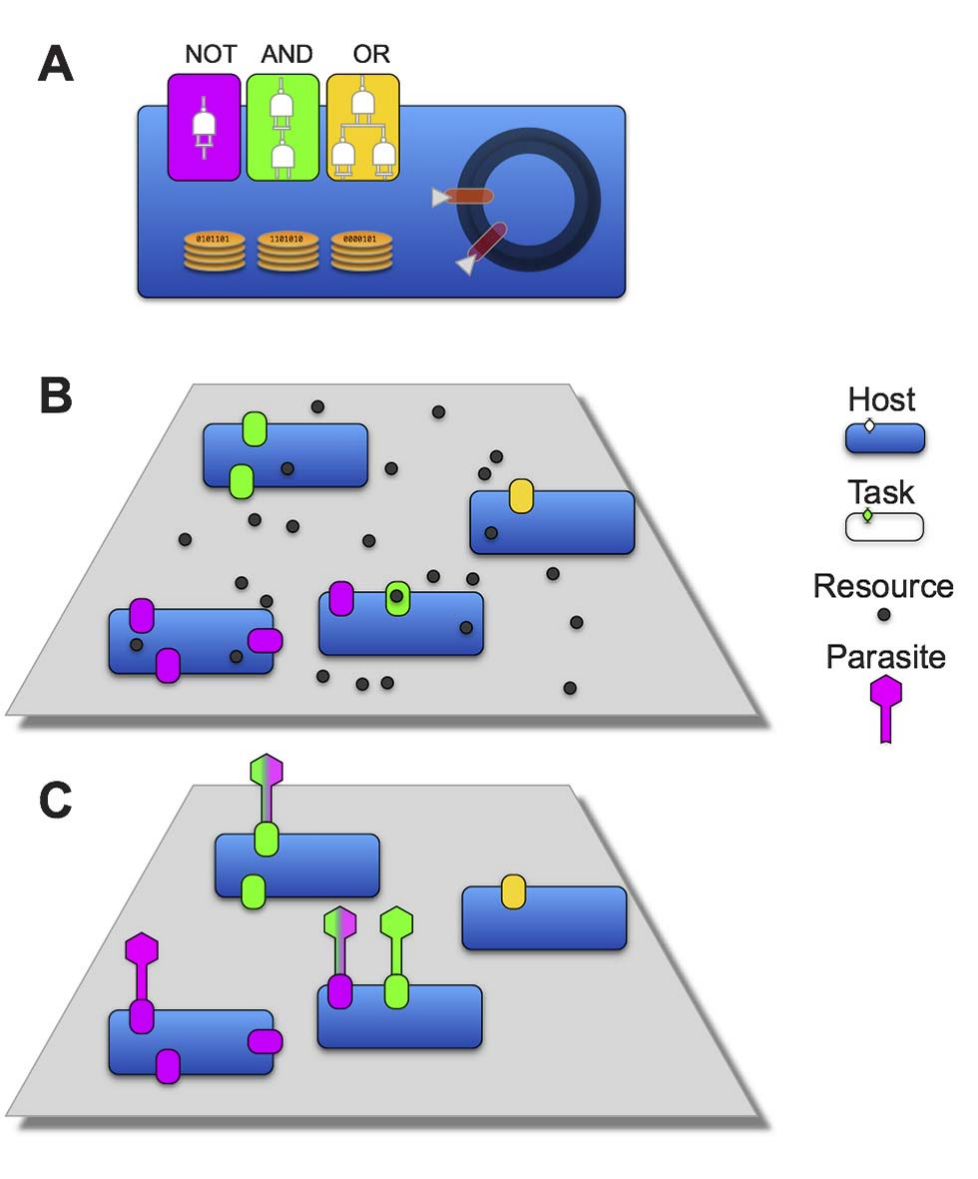
Hosts, parasites, functions, and resources in Avida. (A) A host organism with stacks used to store binary values, a circular genome with pointers used to execute its code, and three functions—NOT, AND, and OR—shown in different colors. Functions vary in complexity as measured by the number of NAND gates (shown as 1, 2, and 3 logic gates within the respective colored function circuits) required to perform them. (B) These functions enable organisms to take up resources from their environment. (C) Parasites target the resource-uptake mechanisms of the hosts in this system by performing the corresponding function. Note that some parasites can perform multiple functions (shown by multiple colors) and thus infect hosts via multiple uptake systems. When a parasite infects a host, it acquires a portion of the host's CPU cycles. Executing a single operation costs an organism a single CPU cycle. Time in these experiments is measured in “updates,” which corresponds to a per capita average of 30 executed CPU cycles.

Figure 1 shows a schematic overview of the relationships between hosts, functions, resources, and parasites in our experiments. Hosts obtain the resources necessary for their reproduction by performing one or more logic functions, but those functions also make the host vulnerable to infection by a parasite that can perform the same function. Thus, an infection can occur only if a particular host and parasite share at least one function, although the specific genetic encoding that a host and parasite employ to perform that function rarely, if ever, correspond at the sequence level. After a successful infection, the parasite acquires 80% of the infected host's CPU cycles, which the parasite uses to execute and copy its own genome, while imposing a severe cost on the host. As a consequence, coevolution occurs when hosts and parasites acquire and lose functions.

The experimental configuration allowed for nine different logic functions, which require varying numbers of NAND instructions to be executed with the proper inputs used for each; NAND is the only logic function available in the genetic instruction set. Although there are many potential measures of functional complexity, the Avida logic environment provides an intuitive metric, as follows. The minimum number of NAND operations required for each function's performance is known and provides a simple, objective measure of the complexity of that function [14]. The most complex function, EQU, requires five NAND operations, and the shortest program that can perform EQU requires nearly 20 precisely interacting instructions, although there are many longer programs that also encode EQU [14]. In the absence of parasites, a previous study found that 23 of 50 populations evolved the ability to perform EQU when the other eight functions were rewarded with additional CPU cycles that increased with their complexity (i.e., minimum required NANDs), thus allowing essential building blocks to accumulate in the evolving genomes [14]. Here, we test whether host-parasite coevolution can drive increased complexity without explicitly rewarding building blocks. To that end, we ran similar experiments except with coevolving parasites in one-half of the replicates and without the progressive reward structure used in the previous work.

## Results and Discussion

### Parasites Drive Greater Host Complexity

**Figure 2:**
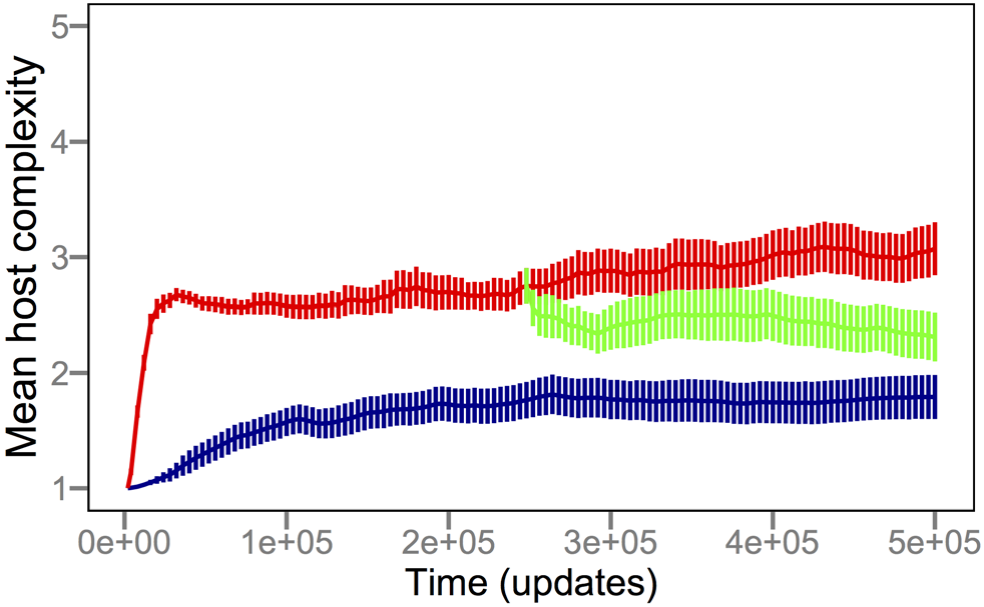
Parasites promote the evolution of host complexity. Complexity was measured as the minimum number of NAND instructions that must be executed by a host to perform its most complex logic function, averaged over all individuals in a population. The blue trajectory shows the grand mean complexity across 50 replicate populations (i.e., runs) that evolved in the absence of parasites. The red trajectory shows the corresponding values for 50 host populations that coevolved with parasites. In 12 runs, the parasites went extinct, in all but one case after 22,000 updates and after the hosts had evolved either the XOR (complexity 4) or EQU (complexity 5) function. The green trajectory shows mean values for the same 50 parasite populations, except here they were “cured” by experimentally eliminating the parasites after 250,000 updates. All populations started with a single host genotype that performs only the NOT function. Updates are arbitrary Avida time units (see Materials and Methods). Error bars are ±2 standard errors of the mean (SEM).

Figure 2 shows that coevolution with parasites drove host populations to evolve more complex functions in order to obtain the resources necessary for their replication, without any greater reward for performing the more difficult functions. Host complexity increased in both the presence (red) and absence (blue) of parasites, but it did so much faster and reached much higher levels in the coevolution treatment (*p*≪0.001, Mann-Whitney *U* = 2,304). The effect of parasites on the rise of complexity is exemplified by EQU, the most complex function; the ability to perform EQU evolved in 17/50 host populations that coevolved with parasites, but in none that evolved without parasites (*p*≪0.001, Fisher's exact test). In a third treatment, parasites were removed at the mid-point of the runs, and the cured host populations (green) evolved substantially reduced complexity relative to the coevolution treatment (*p*≪0.001, Mann-Whitney *U* = 543.5), although the cured hosts retained greater complexity than those that never saw the parasites (*p* = 0.002, Mann-Whitney *U* = 1,703).

The increased complexity relative to the ancestor observed in the absence of parasites (*p*≪0.001, Wilcoxon signed-rank *W* = 1,275) accords with a simple model that couples a random walk in complexity with a selective constraint that limits functional degradation; Gould dubbed this model the “drunkard's walk,” alluding to how a patron leaving a pub eventually stumbles to the curb because the pub itself limits backward movement [5]. In our experiments, all populations started from the same ancestral program that could perform only the simplest function, NOT, and hence they were the least complex programs able to obtain resources and reproduce. Any less complex genotypes generated by mutation could not reproduce and were thus eliminated. More complex organisms also arose by mutation; although they obtained no additional resources for performing more complex functions (and, in fact, might replicate more slowly), they nonetheless could reproduce and thereby persist. Over time, this asymmetrical constraint allowed complexity to increase, albeit slowly and to a limited extent. This explanation of complexity evolving as a “drunkard's walk” does not imply that evolution as a whole operates as a random walk; instead, it only implies that complexity might follow such a pattern.

The coevolutionary process clearly produced greater functional complexity in the hosts. In broad outline, this effect occurs because parasites constantly select for new host phenotypes and thereby cause host populations to explore adaptive landscapes more broadly than hosts that are evolving alone [19]. However, it is not obvious why the effect was so large and continued for so long. Understanding the initial increase in complexity is seemingly straightforward—hosts must evolve some function other than NOT to avoid infection yet still reproduce, and all except one of the other functions have higher complexity than NOT. But this explanation alone cannot explain even the initial step, because the first new function to arise by mutation was, in the vast majority of cases, the one other function, NAND, that also requires executing only a single NAND instruction. In fact, the average complexity of the first new function was only 1.10 (1.01–1.19 95% confidence interval), and the maximum was only 2 in any case. What then might account for the large and sustained rise in complexity? One plausible explanation is an escalatory arms race that gives rise to progressively more extreme and complex adaptations [7],[19],[20]. For example, coevolution between cheetahs and gazelles may have favored ever-increasing speed, which was achieved by evolving more complex musculoskeletal systems. In many systems, however, coevolution does not occur along a single axis, but instead involves many traits [21] and can lead to fluctuating frequency-dependent selection instead of an arms race [22]. For example, such frequency-dependent fluctuations appear to dominate the interactions between *Daphnia magna* and its parasite *Pasteuria ramosa*, as determined by reviving eggs and spores from various sediment depths representing different historical states of the interaction [23].

Escalating arms races and negative frequency-dependent cycling, in general, are the two main outcomes of host-parasite coevolution. Escalation could lead to an increase in complexity if, for example, more complex tasks provided hosts with resistance to any less complex parasites. However, there is no such task “dominance” in Avida. Instead, a particular parasite can infect a particular host provided they share at least one function. Given that requirement, there is no inherent reason that escalation must occur [24],[25]—for example, the host and parasite populations could cycle repeatedly between two states—and so we can reject the arms-race hypothesis as a sufficient explanation for the emergence of more complex traits in hosts that coevolved with parasites. Nonetheless, it is important to note that frequency-dependence and escalation are not mutually exclusive processes.

### Parasites Retain “Memory” of Previous Hosts

**Figure 3:**
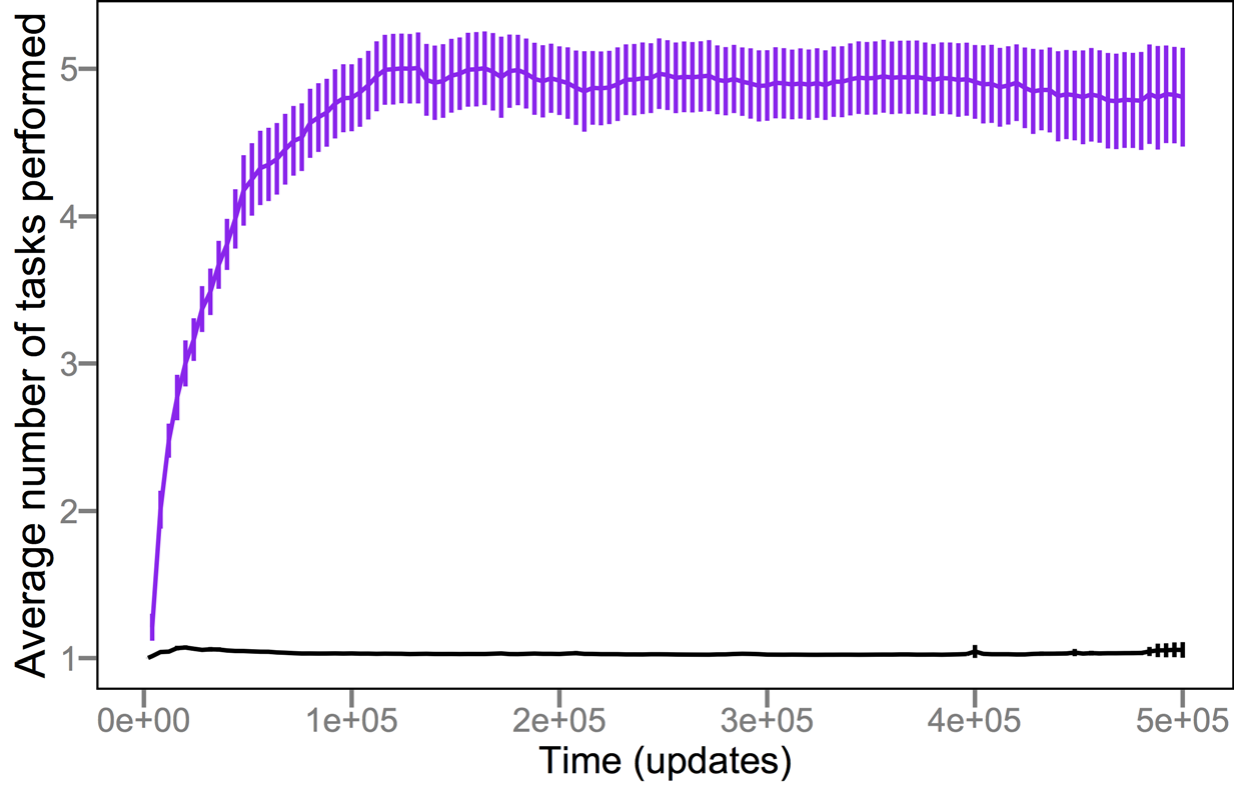
Parasites evolve generalist strategies while hosts remain specialists. The purple trajectory shows the average number of different functions performed by individual parasites across 50 replicates of the coevolution treatment. In 12 cases, the parasite population eventually went extinct, and so the number of replicates declines to 38 over time. The black trajectory shows the corresponding average for individual hosts; host populations were excluded from the average after the corresponding parasite populations had gone extinct. Error bars are ±2 standard errors of the mean (SEM).

How could negative frequency-dependence drive a sustained increase in host complexity rather than producing simple cycles? One possible explanation is that parasites maintain a “memory” of previously encountered host states. If so, then hosts can escape infection only by evolving in a previously unexplored direction—in the Avida system, by evolving an entirely new and therefore usually more complex function to acquire resources, rather than recycling one that was previously discarded after it was targeted by the parasite. The simplest way to achieve such memory is if a parasite population evolves generalist phenotypes that can infect multiple hosts, including types no longer common in the community. Indeed, the coexistence of multiple host types maintained by negative frequency-dependent selection would favor parasites with broad host-ranges. To examine whether this population-genetic memory existed, we quantified the average number of functions that parasites could perform. Consistent with the memory hypothesis, parasites evolved to become generalists that often performed four or five functions and thereby could infect several different host types (Figure 3). By contrast, we expect the hosts to evolve primarily as specialists because an individual needs to perform only one function to obtain resources, and performing multiple functions makes it vulnerable to a broader range of parasites. Indeed, most hosts performed only a single function (Figure 3), although that function became much more complex over time (Figure 2).

To verify that the parasite's population-genetic memory drove the evolution of host complexity, we performed another set of coevolution experiments using a “challenge” design. This design is analogous to a microbiological approach in which bacteria are challenged with phage, a single resistant mutant is isolated, the phage are then challenged with the resistant host, a single host-range mutant is isolated that can overcome the resistance, and the cycle is repeated [26]. Using this design, diversity is lost because only individual mutants are retained at each step, and the advantage to the parasite of retaining a broad host-range (i.e., memory of prior hosts) is reduced or eliminated. Therefore, if the parasite's population-genetic memory drove the evolution of host complexity in the original coevolution treatment (Figure 2), then we expect hosts to achieve reduced complexity under the challenge regime. Indeed, the resulting host complexity was much lower in the challenge treatment than with coevolution (*p*≪0.001, Mann-Whitney *U* = 2,373); in fact, the challenge treatment was indistinguishable from the populations that had evolved without parasites (*p* = 0.43, Mann-Whitney *U* = 1,298).

**Figure 4:**
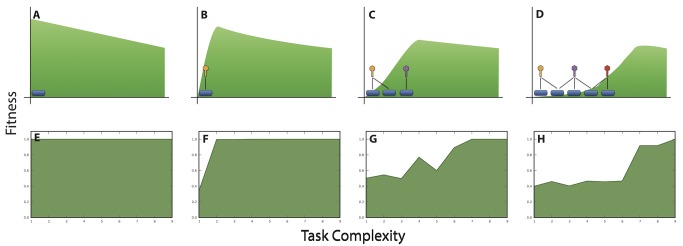
Effects of parasites on the host's adaptive landscape. (A) Assuming that unnecessary complexity is costly in the absence of any direct benefit, the fitness peak corresponds to the simplest host phenotype. (B) Once parasites are introduced, the landscape is deformed and selection favors a more complex host phenotype. (C, D) As coevolution continues, the parasites maintain a population-genetic memory of host phenotypes, which pushes the fitness peak toward higher and higher levels of complexity. (E–H) In the coevolution runs, we quantified the effect of parasites on the host adaptive landscape as the proportion of parasites that were unable to infect hosts performing each of the nine logic functions.

We can form an intuitive understanding of the parasite's population-genetic memory and its effects on the evolution of complexity using the imagery of an adaptive landscape. Consider the case where increasing complexity is disadvantageous because performing more complex functions requires more resources than performing simpler tasks. In the absence of parasites, hosts will evolve the simplest viable functions (Figure 4A). However, when this host is targeted by parasites, the landscape is deformed, creating a new peak at a slightly more complex task (Figure 4B). As coevolution continues, additional hosts and parasites will evolve and a diverse set may be maintained through negative frequency-dependent selection. This community further depresses the landscape, thus moving the peak toward even higher levels of complexity (Figure 4C and 4D). To evaluate whether our experiments supported this intuitive model, we measured the proportion of parasites unable to infect hosts performing each one of the nine logic functions on its own. That proportion represents a critical fitness component of the host because it reflects the host's ability to resist infections by the parasites present in its environment. Figure 4E–4H shows the empirical relationship between average host fitness (i.e., resistance) and the complexity of the task performed over evolutionary time. In support of our population-genetic memory hypothesis, the fitness peak shifted strikingly toward higher levels of complexity as coevolution progressed. Thus, the diversity of parasites—with their individually and collectively broad host-ranges—sustained a memory of previously evolved host phenotypes and generated an adaptive landscape for the host that favored increasingly complex tasks.

### Effects of Breaking the Coevolutionary Feedback

**Figure 5:**
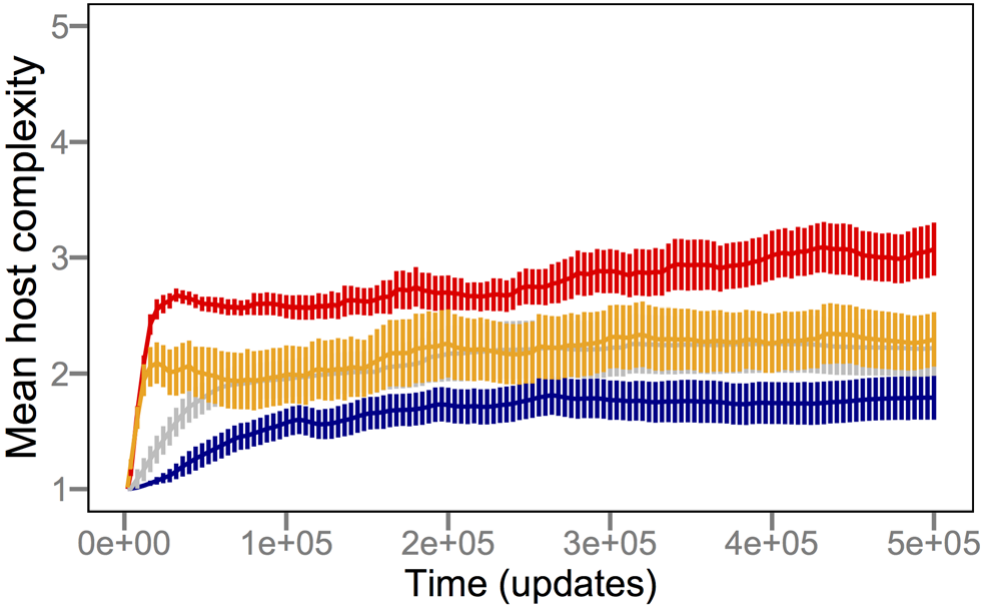
Frozen and replayed parasite genotypes fail to recapitulate the level of complexity seen in the coevolution treatment. Host complexity was measured as in Figure 2, and the coevolved (red) and evolved-without-parasites (blue) treatments are shown as before. The grey trajectory indicates the mean level of host complexity that evolved when parasite genotype frequencies were frozen at the values observed after 250,000 updates of coevolution. The orange trajectory shows the level of complexity that hosts evolved in the replay treatment, where they faced changing, but not coevolving, parasite populations. In this treatment, the parasite genotype frequencies were set to the levels observed during coevolution runs at 1,000-update intervals. The parasites went extinct before 250,000 updates in one of the coevolution replicates, and so the frozen treatment started with 49 replicates. In three of the 49 replicates of the frozen treatment, the hosts overcame the parasites and drove them extinct. In the replay treatment, a total of 30 host populations drove the replayed parasites extinct (including the 12 that went extinct during coevolution). Error bars are ±2 standard errors of the mean (SEM).

To test whether the fitness landscape shaped by a coevolved population of parasites was sufficient to drive the evolution of complexity observed in our original coevolution treatment, we performed a new treatment in which the parasite population began with genotypes “frozen” at the frequency they occurred within each original replicate at 250,000 updates (the halfway point, when the majority of host complexity and parasite diversity had evolved), but further evolution of the parasite was precluded. To maintain constant frequencies of the parasite genotypes, each newly reproduced parasite was assigned a random genotype from the 250,000-update set. After 500,000 updates in this complex-but-static environment of frozen parasite frequencies, hosts evolved significantly higher complexity than in the treatment without parasites (*p* = 0.003, Mann-Whitney *U* = 1,684). However, the hosts confronted with the complex-but-static parasite populations did not reach as high a level of complexity as when the parasites coevolved (*p*≪0.001, Mann-Whitney *U* = 1,946) (Figure 5). This disparity may indicate an effect of fluctuating environments, such that dynamic parasite environments favor increased host complexity more than complex-but-static parasite environments. To test this hypothesis, we then allowed hosts to evolve in environments where we “replayed” the changing parasite genotype frequencies over time from the coevolution treatment, but where these parasite genotypes did not respond to the host evolution that was occurring within any particular replicate. Again, the host populations that evolved in this replay treatment achieved significantly greater complexity than those that evolved without the parasites (*p* = 0.034, Mann-Whitney *U* = 1,529), but the hosts in the replay treatment still did not reach as high levels of complexity as the coevolved hosts (*p*≪0.001, Mann-Whitney *U* = 1,908) (Figure 5).

Thus, coevolved parasites—whether constant (frozen) or varying over time (replayed)—favored the evolution of hosts with more complex functions than hosts that evolved without parasites at all. Nonetheless, the hosts under these treatments failed to evolve the highest level of complexity, which they achieved with coevolving parasites. Coevolution involves reciprocal changes in which the host population influences how the parasite population responds, both ecologically and evolutionarily, and vice versa. Although the parasite population was diverse in both the frozen and replayed treatments, and while it varied in time in the latter treatment, the evolution of the parasite population was decoupled from the evolutionary changes that occurred in the host population. Taken together, these experiments thus indicate that the special push-and-pull of coevolution played a major role in the evolution of host complexity. They also imply a more dynamic view of population-genetic memory, one in which negative frequency-dependence constantly tunes the parasite population in response to host evolution. Without coevolutionary reciprocity, the interactions between host and parasite populations are dissonant and population-genetic memory is ineffective.

### Effects of Starting Condition on Evolved Complexity

**Figure 6:**
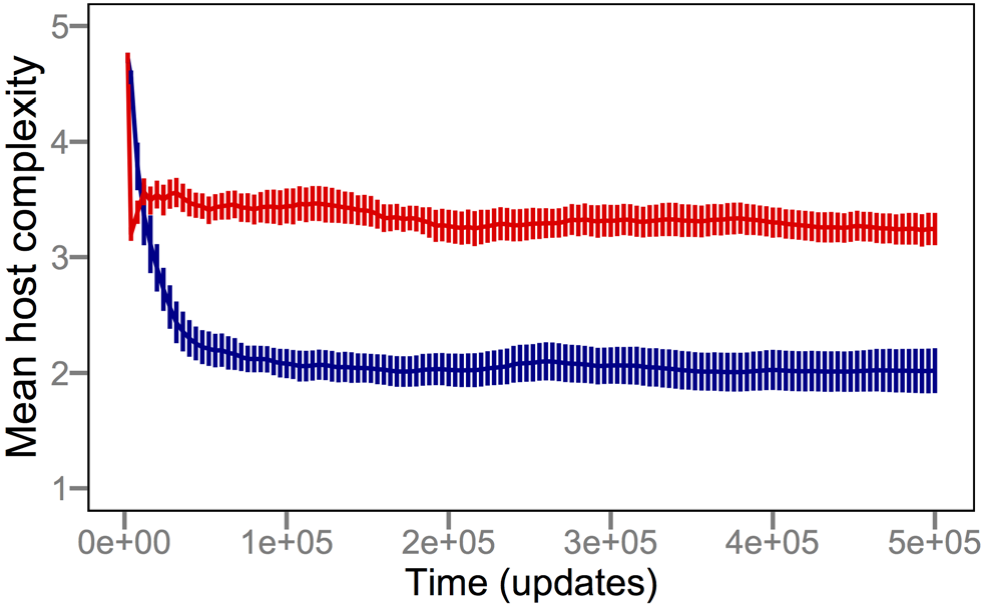
Higher levels of host complexity are also maintained with coevolving parasites when starting from complex ancestors. As in Figure 2, complexity was measured as the minimum number of NAND instructions that must be executed by a host to perform its most complex logic function, averaged over all individuals in a population. The blue trajectory shows the grand mean complexity across 50 replicate populations that evolved in the absence of parasites. The red trajectory shows the corresponding values for 50 host populations that coevolved with parasites. In nine coevolution runs, the parasites went extinct. All populations started with a single host genotype that performed only the EQU function, the most complex task available to them. Error bars are ±2 standard errors of the mean (SEM).

We started the previous experiments with hosts and parasites capable of performing only the simplest logic functions in order to understand how coevolution might drive the emergence of complex functions from simpler ones. However, we can also ask whether coevolution would sustain the greater functional complexity if the experiments began with hosts and parasites that could perform the most complex function, EQU. Indeed, coevolution maintains much higher complexity than evolution alone from this alternative starting point (Figure 6). In these runs, host complexity initially declined rapidly when the parasites were present because the hosts readily escaped by performing new, simpler, tasks. However, as the hosts exhausted the simple tasks that were easily evolved, their complexity leveled off at a higher value than without parasites (*p*≪0.001, Mann-Whitney *U* = 2,305) (Figure 6). Although the average complexity across populations never dropped below ∼3, genotypes within the host populations explored the simplest functions (Figure S1). In all but one population, the frequency of hosts that performed only the simplest tasks transiently exceeded 10%. The apparent equilibrium levels of host complexity with and without coevolving parasites were evidently the same whether the experiments began at low or high complexity (compare Figures 2 and 6).

### Effects of Coevolving Parasites on Host Phylogeny

**Figure 7:**
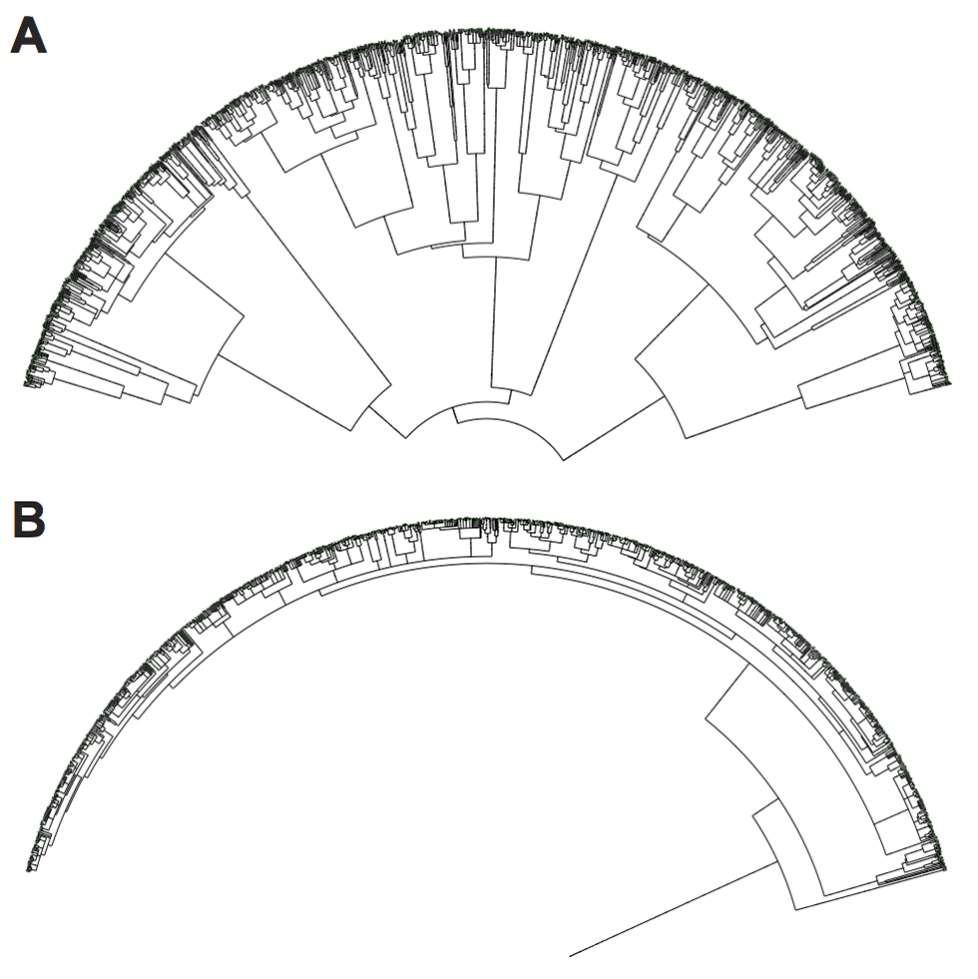
Effect of coevolving parasites on host phylogenies. Representative phylogenies for hosts that evolved in the (A) presence and (B) absence of parasites. The branch leading to the original ancestor is too short to be seen in (A). The phylogenies show all of the host genotypes present at the end of the run, and the phylogenies are known exactly in this system.

**Figure 8:**
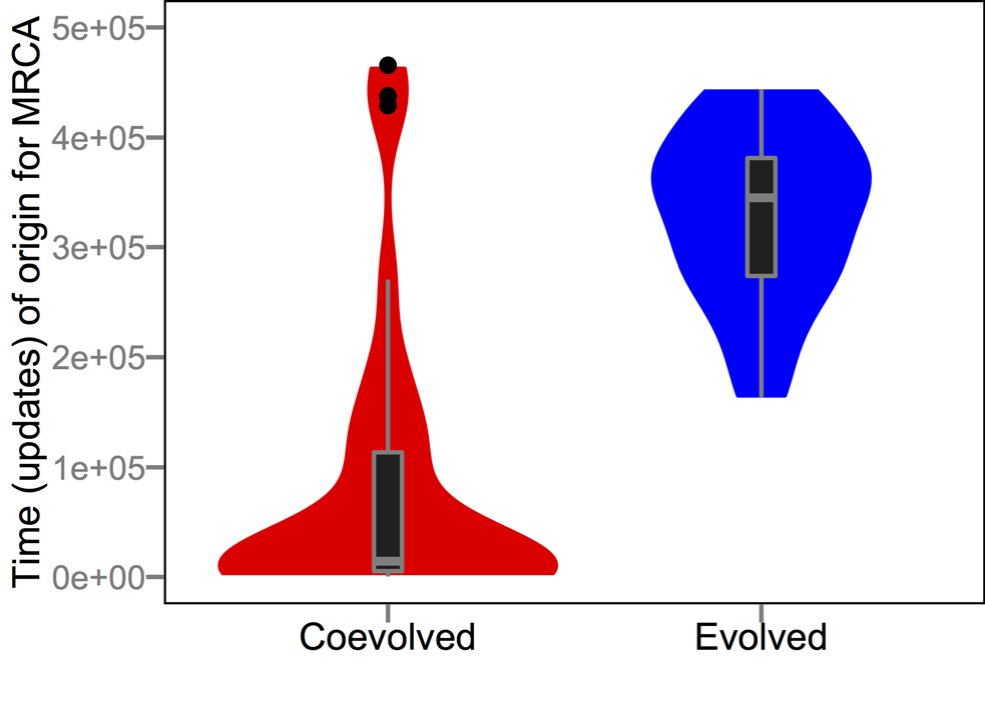
Effect of coevolution on coalescence times in host phylogenies. The data are shown as box plots and smoothed frequency distributions for the times of origin of the MRCA in 38 host populations that coevolved with parasites (excluding the 12 runs where the parasites went extinct) and 50 populations that evolved without parasites. The MRCAs arose significantly earlier in the coevolution treatment. The tail of the distribution for the coevolution treatment is more pronounced if we include the host populations where the parasites went extinct, but the difference remains highly significant (*p*≪0.001, Mann-Whitney *U* = 1,859). Box hinges depict first and third quartiles and whiskers extend 1.5× interquartile range (IQR) out from their corresponding hinge.

Coevolution with parasites also had profound effects on the phylogenetic structure of host populations and on the phenotypic evolvability of host genomes. With respect to phylogenies, the frequency-dependent nature of host-parasite interactions promotes not only greater diversity at any given moment but also deeper branches that reflect the preservation of diversity through time. In Avida, we can track genealogies precisely and thus construct exact phylogenetic trees, avoiding uncertainty about historical states and branch lengths. Figure 7 shows representative trees for host populations that evolved in the presence and absence of parasites, and they differ strikingly in their coalescence profiles. To formalize this difference, we calculated the time since the most recent common ancestor (MRCA) for all 50 host populations in the coevolution and evolution-without-parasites treatments (Figure 8). The MRCA in coevolved host populations usually arose soon after the experiment began (median 3% of the total elapsed time), whereas the MRCA in the absence of parasites typically dated to well after the midpoint (median 70%), and this difference is highly significant (*p*≪0.001, Mann-Whitney *U* = 1,742). Thus, coevolution not only affects the outcome of adaptation, but also fundamentally changes how those outcomes are reached. Coevolution was similarly found to increase the rate of adaptation when embedded in multispecies networks of mutualists [27]. Although the systems and form of interactions are different, their similar results suggest the important role reciprocity plays in evolving systems.

### Effects of Coevolving Parasites on Host Evolvability

**Figure 9:**
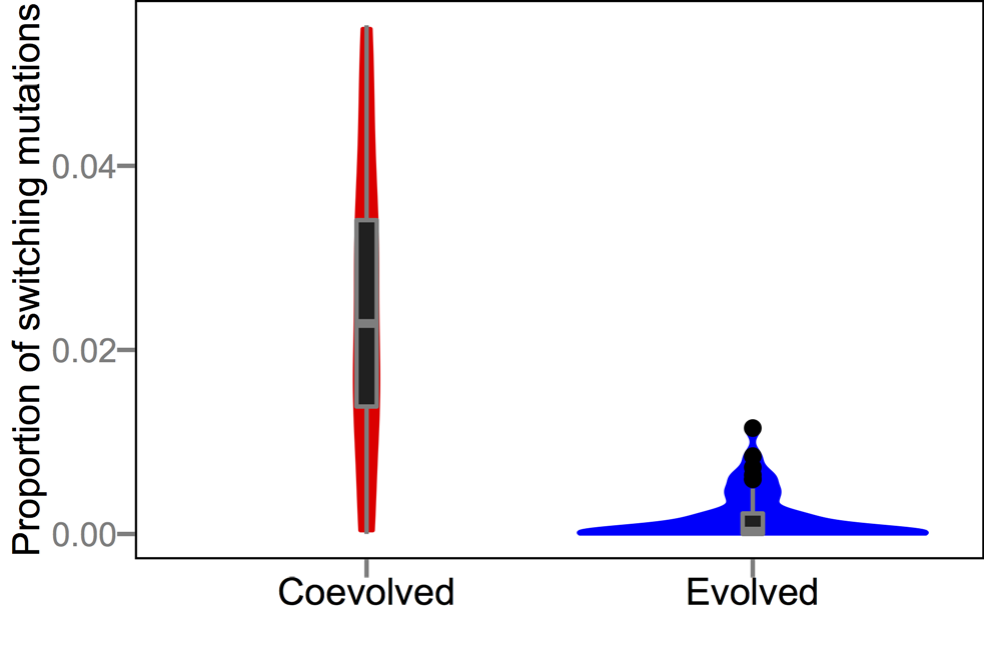
Proportion of point mutations in host genomes that switch functions without changing the number of functions performed. The data are shown as box plots and smoothed frequency distributions. Proportions were obtained by testing all possible one-step point mutations in the genetic background of the most abundant host genotype at the end of all 50 runs with and without parasites. Box hinges depict first and third quartiles and whiskers extend 1.5×interquartile range (IQR) out from their corresponding hinge.

**Figure 10:**
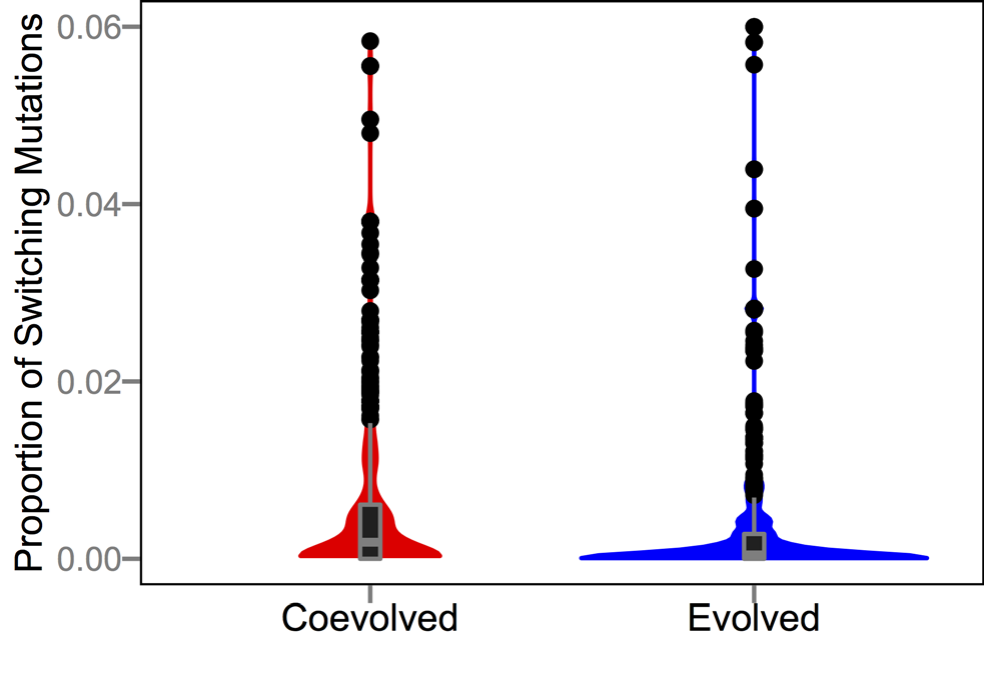
Proportion of point mutations that switch functions without changing the number of tasks performed in paired genotypes, where each pair includes hosts from the coevolution and evolution treatments that perform identical sets of tasks. Proportions were obtained by testing all possible one-step point mutations in each genetic background. Box hinges depict first and third quartiles and whiskers extend 1.5×interquartile range (IQR) out from their corresponding hinge.

Previous research using Avida showed that different treatments could drive populations into qualitatively different regions of the fitness landscape; specifically, populations that experienced higher mutation rates evolved onto lower but flatter regions of genotypic space than populations that evolved at lower mutation rates, a phenomenon dubbed “survival of the flattest” [28]. Here we examine whether coevolution with parasites produced host genomes that were more evolvable with respect to escaping infections. To that end, we mapped phenotypic changes onto every possible one-step point mutation for the most common host genotype from all evolved and coevolved populations at the end of the experiment. Several types of phenotypic changes are possible including the gain of a function, the loss of a function, or switching which function is performed without changing the total number of functions performed. Mutations in the last category are of particular interest because, in the presence of parasites, the ability to switch functions without requiring intermediate steps (adding a new function before losing the old one) could be adaptive. That is, more evolvable hosts would be able to change phenotypes faster and could thereby escape coevolving parasites more readily. While selection does not directly favor hosts with more evolvable genotypes, they are more likely to produce surviving lineages when coevolving with parasites; thus, second-order selection could drive the evolution of evolvability. In strong support of this hypothesis, function-switching mutations were >10-fold more common in hosts that evolved with parasites than in hosts that evolved without parasites (*p*≪0.001, Mann-Whitney *U* = 2,338) (Figure 9). To evaluate whether this effect might somehow merely reflect the more complex tasks typically performed by coevolved hosts, we analyzed pairs of genotypes from the coevolved and evolved host populations that perform identical sets of tasks. The coevolved hosts were still significantly more evolvable than their paired evolved host (*P*≪0.001, Wilcoxon signed-rank *W* = 112,616.5) (Figure 10), although the frequency of task-switching mutations tended to be lower in both treatments after this pairing procedure. Thus, coevolution drove host populations to occupy more evolvable regions of the adaptive landscape.

Taken together, our experiments show that parasites pushed hosts to levels of functional complexity that were well beyond what they achieved by random walks (Figure 2). This complexity resulted from population-level processes [29]–[31], in which frequency-dependent interactions sustained generalist parasites (Figure 3) that were supported by phenotypically and phylogenetically diverse hosts (Figures 7 and 8). If population-level effects were eliminated, as in the challenge experiments, then host complexity remained low. Moreover, if the coevolutionary feedback between hosts and parasites was broken by freezing or replaying parasite genotypes, then hosts did not evolve such complex tasks as when parasite populations could respond to the changing host population (Figures 4 and 5). Although the form of interactions between the hosts, their resources, and parasites in our study system (Figure 1) strongly constrained host evolution (e.g., hosts performing multiple functions were more broadly susceptible to parasites and rarely observed), hosts nevertheless overcame these limitations by becoming more evolvable (Figure 9). In particular, host genomes evolved such that a much larger proportion of mutations caused a switch from one resource-acquisition function to another, thereby allowing hosts to escape, in a single step, parasites that targeted the first function. These results—from an unusual but highly tractable system—add to growing evidence from experiments and theory that coevolutionary processes promote biological diversity, new functions, and evolvability [16],[17],[20]–[25],[29]–[35].

## Materials and Methods

### Evolution Experiments

All experiments were performed using the Avida 2.13.0 software, which is available without cost (http://avida.devosoft.org/). Configuration files with the parameter settings used and data files have been deposited into the Dryad Repository: http://dx.doi.org/10.5061/dryad.485qq [36]. Host and parasite populations lived in a well-mixed chemostat-like environment, with a single type of resource entering at a constant rate. Hosts obtained resources required for replication by performing any of nine distinct one- and two-input logic functions, provided there were resources available in the environment. A parasite could infect a host if they performed at least one function in common, and an infecting parasite then acquired 80% of its host's energy (CPU cycles) [37]. The ancestral hosts and parasites could perform only NOT, one of the two simplest functions. We initially monitored evolution under two main treatments, each with 50-fold replication: host organisms evolved alone in one treatment, and they coevolved with parasites in the other. Each replicate started with a different numerical seed, and the resulting sequence of pseudo-random numbers influenced mutations, parasite-host encounters, and other probabilistic events. The parasites went extinct in 12 coevolution runs; except where otherwise noted, we included those runs in our analyses. In a third treatment, the parasites were experimentally removed halfway through each run, with the first half being identical to a run in the coevolution treatment (i.e., using the same initial seed).

All runs lasted for 500,000 updates; an update is an absolute time unit in Avida equal to the execution, on average, of 30 instructions per individual host organism. Generation times for the ancestral host and parasite genotypes were 63 and 23 updates, respectively, although generation times changed as genomes evolved. Each host population began with one individual; the carrying capacity was 14,400 in the absence of parasites. In the coevolution treatment, 400 parasites were introduced after 2,000 updates; only a single parasite could infect an individual host. Mutation rates were 0.25 and 0.5 per genome replication for the ancestral host and parasite, respectively, of which 90% were point mutations and 5% each were insertions or deletions of single instructions. Per-site mutation rates were constant, so total genomic rates varied with changes in genome length. Mutations occurred at random with respect to genome position.

### Challenge Experiment

To eliminate all population-level interactions in both species, we screened individual hosts and parasites for defenses and counter-defenses, rather than using evolving populations. Starting from the same ancestral host, we generated thousands of individuals using the same mutation regime as in the evolution experiments, and we randomly chose a single host mutant that was resistant to the ancestral parasite. We then repeated this process for parasites, again using the same mutation regime as in the evolution experiments, and we isolated a host-range mutant able to infect that resistant host mutant. We continued the pairwise challenges using the derived host and parasite genotypes for 50 rounds. A challenge experiment was stopped if we failed to isolate a relevant mutant after screening 500,000 individuals. In the comparisons with the evolution and coevolution treatments, we used 56 challenge experiments (out of 100 started) that achieved the full 50 rounds of reciprocal defenses and counter-defenses. However, the truncated runs appeared to be indistinguishable from those that went the full duration.

### Freeze and Replay Experiments

In these experiments, we allowed host populations to evolve with either “frozen” or “replayed” parasites. During the original coevolution experiments, we saved each replicate's entire set of host and parasite genotypes every 1,000 updates. We modified the Avida source code such that this record of genotypes can be loaded into an on-going run at any point by adding an option to override the normal replication process with one that samples from a genotype list. When organisms reproduce, instead of inheriting their parent's genome, the offspring is assigned a random genotype from the list. This procedure can be implemented for hosts, parasites, or both; however, in the freeze and replay experiments presented here, we manipulated only the parasite populations using this new procedure. In both treatments, we injected 1,500 parasites into the host population after 2,000 updates; this number was increased relative to the coevolution treatment to ensure that the frozen and replayed parasite populations, which were sometimes poorly adapted to the ancestral host, did not go extinct.

In the freeze treatment, each host population confronted a parasite population that was complex and diverse, but constant in its genotypic frequencies over an entire run (except for the fluctuations associated with births and deaths of the parasites). The composition of each parasite population was based on the list of parasites taken at the mid-point (i.e., 250,000 updates) of one of the coevolution treatment runs. Thus, the genetic composition of the parasite population was frozen throughout the run, although the total number of parasites could rise or fall in accord with the dynamics of infections. Under the replay treatment, the frequencies of parasite genotypes changed over time, but those changes were based on parasite evolution that had taken place in an earlier coevolution run, rather than on the dynamics that were occurring in the replay itself. That is, the list of parasite genotypes from which new parasites were drawn was changed every 1,000 updates to reflect what had happened in the earlier run. As a consequence, the host could evolve in response to the changing frequencies of the various parasite genotypes, but not vice versa—the coevolutionary feedback was broken, although parasite diversity and the temporal changes in that diversity were preserved.

### Phylogenetic Analysis

In Avida, the genealogy of organisms is known perfectly and, when coupled with the asexual lineages studied here, allows construction of the exact phylogenetic history for a population. We used the python ete2.1 module to represent (Figure 7) all of the genotypes present in two host populations along with their ancestries through the various coalescences, the most recent common ancestor for the entire population, and the founding genotype.

### Evolvability Analysis

We tested every possible one-step point mutation in the genetic background of the most abundant host genotype at the end of all 50 evolution and coevolution runs. Each mutant was placed into one of the following categories based on the phenotypic changes relative to its parent: (i) the mutant cannot perform any functions or is otherwise nonviable; or (ii) the mutant is viable and (a) there is no difference in the number or identity of functions performed; (b) the mutant performs more functions; (c) the mutant performs fewer functions; or (d) the identity of functions performed has changed, but the number has not. The last category, which we call “switching,” was the focus of our analysis.

We also modified this analysis to take into account possible effects of differences in the number and complexity of tasks performed by pairing host genotypes isolated from the evolved and coevolved populations that performed identical sets of tasks. Genotypes were pooled across the replicate runs based on what tasks they could perform. All of the coevolved populations were compared with all of the evolved populations to identify paired host phenotypes that performed identical sets of tasks. For each pair of phenotypes thus identified, a genotype from the evolved and coevolved populations that performed the appropriate set of tasks was chosen at random, and all possible one-step mutations were then generated for both genotypes.

## Supporting Information

Figure S1 **Proportion of hosts at all five levels of complexity over the first 25,000 updates in each of the 50 replicates seeded with the most complex ancestors.** White regions represent host genotypes that performed only the simplest tasks, while progressively darker regions represent hosts that performed more complex functions. All of the host populations transiently harbored subpopulations that could perform only the simplest functions, although coevolving parasites drove the host populations to perform more complex functions on average (Figure 6).
(TIF)

## Abbreviations

MRCA: most recent common ancestor
