# Supplementary figures and images for: Coevolution Drives the Emergence of Complex Traits and Promotes Evolvability
Source: PLoS Biol. 2014 Dec 16;12(12):e1002023. doi: 10.1371/journal.pbio.1002023 (PMC4267771; doi:10.1371/journal.pbio.1002023)

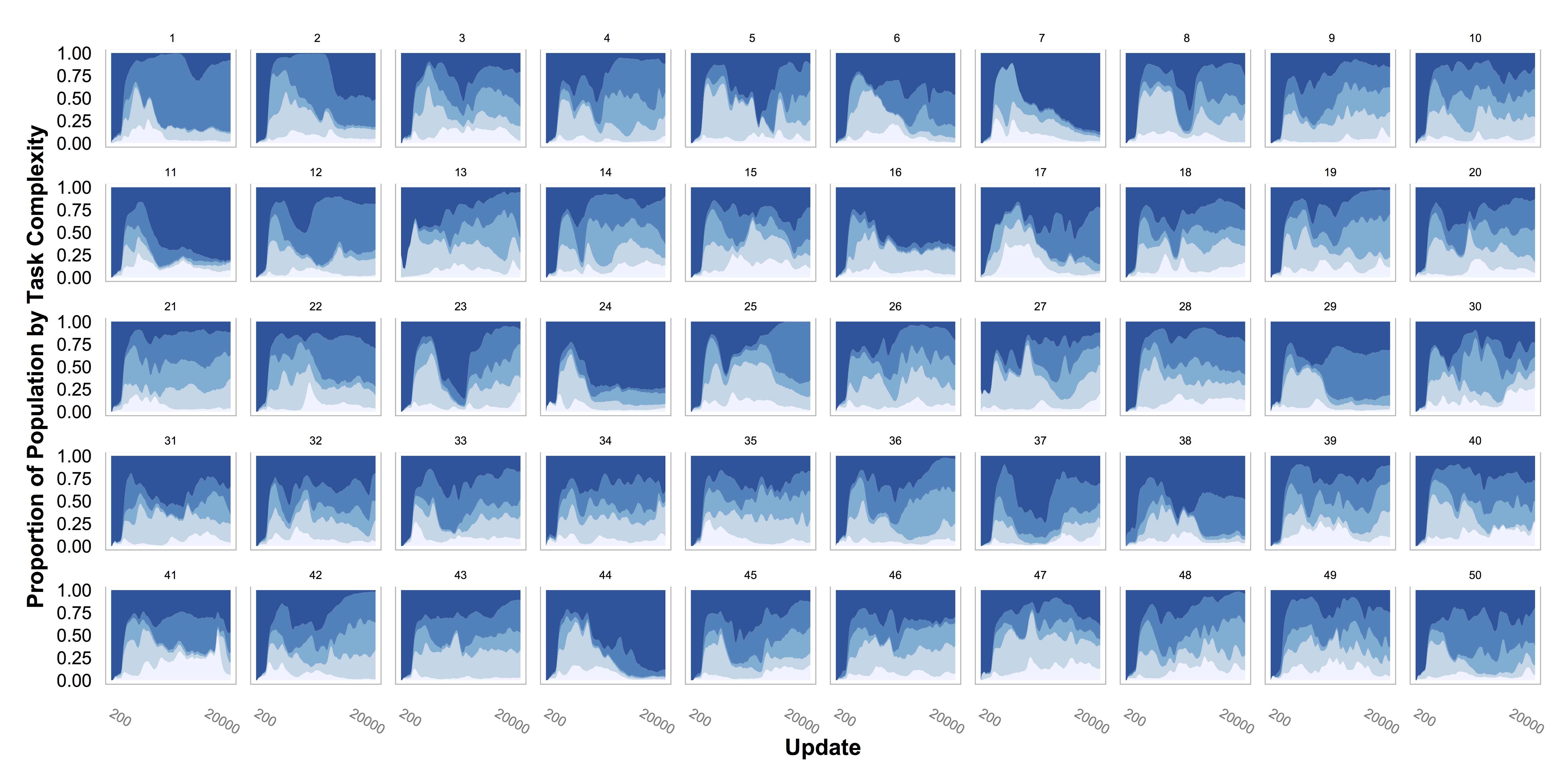

Supplement: Figure S1 — Proportion of hosts at all five levels of complexity over the first 25,000 updates in each of the 50 replicates seeded with the most complex ancestors. White regions represent host genotypes that performed only the simplest tasks, while progressively darker regions represent hosts that performed more complex functions. All of the host populations transiently harbored subpopulations that could perform only the simplest functions, although coevolving parasites drove the host populations to perform more complex functions on average (Figure 6). (TIF) [file pbio.1002023.s001.tif]
